# Trials for Offences against Health

**Published:** 1855

**Authors:** 


### TRIALS FOR OFFENCES AGAINST HEALTH.

SEVERAL trials have lately taken place for nuisances, filthy lodging-houses, and other similar offences. The following case is the most flagrant one recently recorded. We reprint it to shew to what extent these atrocities prevail in London and other large towns:—

John Lyons, an Irishman, who keeps a common lodging-house which he has hitherto neglected to register, at 31, Farmer Street, Shadwell, appeared before Mr. Ingham, to answer a summons taken out by Messrs. Reason and Price, the inspectors appointed to carry out the provisions of the act, for the regulation of common lodging-houses, and which charged the defendant with refusing to comply with the provisions of the law, and with keeping a most filthy and unwholesome establishment. The disclosures made were quite startling, and the defendant's house may be considered as a fair sample of the horrible dens in which Lascars and Chinamen were lodged, until Messrs. Reason and Price interfered with them.

James Kirby, 53 A, a police sergeant, and inspector of common lodging-houses, stated that on Saturday night last, he visited the defendant's house, which was in a most filthy and dilapidated condition. In the first room, he discovered a Chinaman sleeping in a cupboard or small closet, filled with cobwebs. The wretched creature was without a shirt, and was covered with a few rags. The Chinaman was apparently in a dying state, and had since expired. An inquest was held on his remains, and it was proved he had died of fever, and had been most grossly neglected. The room in which the Chinaman lay was destitute of bedding and furniture. In the second room he found Aby Callaghan, a poor Irish widow, who said she paid a rent of 1s. 6d. per week. In the third room was Abdallah, a Lascar, who said he paid 3s. per week. In the same room were two prostitutes, almost in a state of nudity, and a Chinaman squatting on a chair smoking. In the fourth room was Dong Yoke, a Chinaman, who said he paid 2s. 6d. per week, for the privilege of sleeping on the bare boards, two Lascars on bedsteads smoking opium, and the dead body of a Lascar, lying on the floor, and covered with an old rug. In the fifth room was an Asiatic seaman named Peru, who said he paid 3s. per week, and eleven other Lascars, six of whom were sleeping on bedsteads, three on the floor, and two on chairs. If the house were registered, only four persons would be allowed in the room. The effluvium caused by smoking opium, and the overcrowded state of the room, was most nauseous and intolerable. In the kitchen, which was very damp, he found Sedgoo, who said he had to pay 2s. per week, and eight Chinamen huddled together. The stench here was very bad. If the house were registered, no one would have been allowed to inhabit the kitchen at all. He should say the house was quite unfit for a human habitation. The floors of the rooms, the stairs and passages were in a filthy and dilapidated condition, covered with slime, dirt, excrement, and all kinds of odious substances. Before the house could be registered it must be limewashed and repaired, water laid on, the yard paved, retiring places constructed, and bedding and bed-linen provided.

In answer to Mr. Ingham, Kirby said he caused the dying Chinaman to be removed in a cradle to the workhouse, where he expired.

Inspector Price said, he had given notice to the defendant to register his house, and comply with the requirements of the act, but he had obstinately refused to do so. The defendant was fined 40s. on a similar information on the 9th of January, and in default of payment, committed for fourteen days.

Mr. Ingham.—Yes, I know he has been had up several times for nuisances and other matters.

Mr. Price.—There have been many cases of fever in the defendant's house.

Mr. Ingham.—I don't wonder at it. The house is a most abominable nuisance, and I shall now fine the defendant £5, and in default of payment, one month's imprisonment.

Several other summonses against the keepers of common lodging-houses were heard; and after evidence from Messrs. Reason and Price, and from Sergeants Maddigan and Kirby, Nos. 51 and 53 A, the magistrate convicted the defendants in various penalties.—*Times, February* 19, 1855.

---

